# Repetition suppression: a means to index neural representations using BOLD?

**DOI:** 10.1098/rstb.2015.0355

**Published:** 2016-10-05

**Authors:** Helen C. Barron, Mona M. Garvert, Timothy E. J. Behrens

**Affiliations:** 1 MRC Brain Network Dynamics Unit, Department of Pharmacology, University of Oxford, Mansfield Road, Oxford OX1 3TH, UK; 2 Oxford Centre for Functional MRI of the Brain, Nuffield Department of Clinical Neurosciences, University of Oxford, John Radcliffe Hospital, Oxford OX3 9DU, UK; 3 Wellcome Trust Centre for Neuroimaging, Institute of Neurology, University College London, London WC1N 3BG, UK

**Keywords:** repetition suppression, functional magnetic resonance imaging adaptation, neural representation, neural computation

## Abstract

Understanding how the human brain gives rise to complex cognitive processes remains one of the biggest challenges of contemporary neuroscience. While invasive recording in animal models can provide insight into neural processes that are conserved across species, our understanding of cognition more broadly relies upon investigation of the human brain itself. There is therefore an imperative to establish non-invasive tools that allow human brain activity to be measured at high spatial and temporal resolution. In recent years, various attempts have been made to refine the coarse signal available in functional magnetic resonance imaging (fMRI), providing a means to investigate neural activity at the meso-scale, i.e. at the level of neural populations. The most widely used techniques include repetition suppression and multivariate pattern analysis. Human neuroscience can now use these techniques to investigate how representations are encoded across neural populations and transformed by relevant computations. Here, we review the physiological basis, applications and limitations of fMRI repetition suppression with a brief comparison to multivariate techniques. By doing so, we show how fMRI repetition suppression holds promise as a tool to reveal complex neural mechanisms that underlie human cognitive function.

This article is part of the themed issue ‘Interpreting BOLD: a dialogue between cognitive and cellular neuroscience’.

## 1. Introduction

Neural activity is responsible for our perception, thoughts and ideas, and the behaviours that we execute. However, the means by which the brain uses neural activity to encode and translate information into complex cognitive processes remains one of the most important challenges for contemporary neuroscience. Over the last few decades, large-scale electrophysiological recordings in animal models have allowed for descriptions of neural activity at high spatio-temporal resolution. This has provided important insight into some of the underlying principles and mechanisms of neural coding, allowing representations to be reasonably well characterized and computations inferred. For example, large-scale recordings have contributed to our understanding of the population dynamics underlying motor responses [1], choice [2] and memory consolidation [3]. In addition, methods of perturbation (such as optogenetics) and sophisticated correlational analyses have together been used to further establish the neural circuit mechanisms that underlie behavioural control [4–6].

However, such invasive recording is largely restricted to investigation in animal models, except under unusual circumstances such as pre-operative recording in epilepsy patients [7,8]. This limits its utility as a tool to understand the human brain because we cannot necessarily assume that information is encoded in the same manner across species, nor ignore the contribution of brain regions that exist in humans but may be absent in other species. These issues are therefore particularly pertinent when investigating complex cognitive processes and neuropsychiatric pathology, which cannot be modelled in animals.

Rather, we must develop ways to indirectly measure neural activity in the human brain using non-invasive techniques. Functional magnetic resonance imaging (fMRI) constitutes one of the principal tools for recording neural activity non-invasively in humans. Compared with other non-invasive recording techniques, such as electroencephalography (EEG) or magnetoencephalography (MEG), fMRI allows for relatively high spatial resolution measurements of human brain activity. It can therefore be used to localize neural activity to particular brain regions and map specialized psychological functions, such as face-, body- and place-related processing [9–11]. More recently, model-based fMRI studies have been used to identify signatures of neural activity, which contribute to particular computations, such as reward prediction error [12] and value computations [13–15].

While these seminal fMRI studies have provided insight into the functional specialization of areas within the human brain, our ability to directly measure the response of individual neurons is severely compromised by the fact that a typical 3 mm^3^ voxel contains more than 10^5^ neurons. Together these neurons contribute to the average activation profile of a voxel, making it difficult to infer the functions and computations performed by subpopulations of neurons. Nevertheless, there are now well-validated strategies that can be used to refine the coarse resolution of the fMRI signal which provide a means to investigate neural activity at the meso-scale, the level of neural representations.

Two fMRI techniques are currently being widely used to access neural information in humans at a more precise spatial resolution, namely repetition suppression and multivariate pattern analysis. fMRI repetition suppression (also termed fMRI adaptation) relies on the fact that neurons show suppression in their response to repeated presentation of stimuli or information to which they are sensitive (see the following for existing reviews: [16–20]). This phenomenon is robustly observed across brain regions and species, in wake and sleep, and using a range of different measurement techniques.

fMRI multivariate pattern analysis (MVPA), on the other hand, takes advantage of small biases in the distribution of functionally specific neurons across neighbouring voxels. Such biases give rise to variation in the activation pattern across voxels, which can be used to infer underlying neural representations (see the following for existing reviews: [21–24]). Multivariate approaches are powerful when applied to brain regions such as visual and temporal cortex where neurons with similar functional selectivity are organized into cortical columns of several hundred micrometres of width [25–27]. The uneven distribution of functionally selective columns across neighbouring voxels, or ‘clustering’ [28], provides a coarse activation pattern, which allows visual features to be successfully classified.

However, not all brain regions display a columnar organization or show an uneven distribution of functionally selective neurons across neighbouring voxels. Regions such as the prefrontal cortex, which lack large columnar organization [29] and show heterogeneous neural response profiles with nonlinear, mixed-selectivity to various task features [30], may be less amenable to MVPA. Nevertheless, multivariate techniques have been used to decode complex brain states [31] and memory retrieval [32] from frontal brain areas.

There are currently few direct comparisons between fMRI adaptation and MVPA, however those investigators that have used both methods in the same experimental paradigm suggest that the two approaches are highly correlated [33,34]. When assessing the relative merits of these two techniques, it is worth remembering that they each measure different aspects of the BOLD signal, and consequently differ in their sensitivity to particular features of the neural code [34]. While repetition suppression has a direct neurophysiological correlate which can facilitate comparison with electrophysiological measures of neural activity, MVPA can provide greater sensitivity when decoding neural representations, particularly in situations where the response selectivity of neurons introduces fine-grained microstructure across voxels.

In this review, we focus on fMRI repetition suppression as a tool to infer neural representations in the human brain. We provide a detailed account of the relationship between single neuron activity and fMRI repetition suppression, and assess evidence for the underlying mechanisms that support the phenomenon. We then discuss the merits and limitations of using fMRI repetition suppression to infer neural computations in the human brain before discussing implications for experimental design.

## 2. Characterizing repetition suppression at a single-cell level

**Figure 1.**
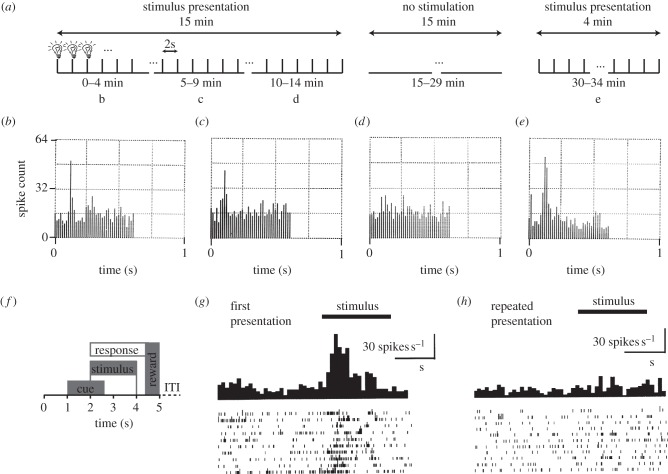
Suppression in neural activity in response to repeated stimuli. (*a*) Experimental timeline of suppression experiment reported in [35]. From 0 to 15 min a 9 µs light stimulus was presented every 2 s. From 15 to 30 min it was switched off, before flashing was resumed at the same frequency from 30 to 34 min. Panels (*b*– *e*) show post-stimulus histograms of a single neuron in the primate IT cortex over the 1 s period following the onset of a light stimulus, at different times during the experiment: (*b*) 0–4 min; (*c*) 5–9 min; (*d*) 10–14 min; (*e*) 30–34 min, adapted from [35]. (*f*) Timeline of a trial in the serial recognition task used to probe recency responses in the macaque entorhinal cortex. Pictures of naturalistic scenes or objects were presented in random order, with a variable number of pictures between the repetition of any given picture. In every trial, the animal indicated by button press whether a picture was novel or familiar. Correct responses were rewarded. (*g*) Responses of neurons in the entorhinal cortex to the first presentation of a novel picture, shown as peristimulus histograms and raster plots for 10 trials. Bin width is equal to 100 ms. (*h*) Responses of neurons in the entorhinal cortex when a novel picture is repeated. Panels (*f*–*h*) adapted from [36].

Repetition suppression was first reported from single-unit recordings in the primate inferotemporal (IT) cortex [35]. In response to repeated presentation of a light stimulus (figure 1*a*), neurons in the IT cortex initially showed a large response (figure 1*b*), but with repeated exposure to the stimulus the response waned (figure 1*c*–*d*). When the stimulus was presented again, after it had been turned off for 15 min, the response of the neuron was fully recovered (figure 1*e*).

**Figure 2.**
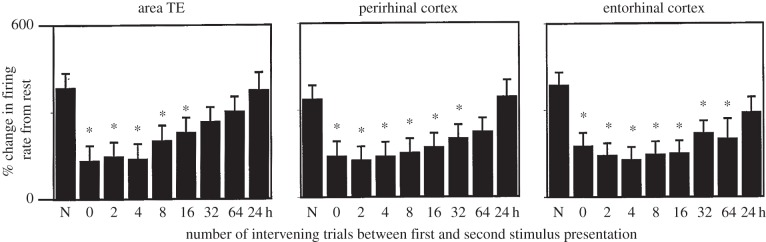
Effect of interleaving stimuli on repetition suppression. Mean percentage change in neuron response to visual stimulus presentation relative to spontaneous activity as a function of the number of interleaved stimuli in (*a*) area TE, (*b*) perirhinal cortex, and (*c*) entorhinal cortex. With an increasing number of intervening trials between the first and second presentation of the stimulus, a decrease in the repetition suppression effect is observed. Asterisk indicates significant repetition suppression effect. N: first presentation of a stimulus. Adapted from [36].

Repetition suppression has since been observed in single-unit activity across a large number of different experimental conditions and brain regions, including the IT cortex [35,37–39], V1 [40–44], somatosensory cortex [45], prefrontal cortex (PFC) [46,47], rhinal cortex [48], entorhinal and perirhinal cortex ([36], see figure 1*f*–*h* for example repetition suppression in the entorhinal cortex). Repetition suppression can occur across a range of repetition time lags, including when multiple interleaving stimuli are presented in between the repeating stimulus [36,49] (figure 2). In a small portion of neurons, the repetition suppression has been observed for a time-lag of up to 24 h [36].

Repetition suppression therefore appears to be a general property of neurons. Suppression occurs relative to how recently a stimulus was presented and can therefore be described as an automatic short-term memory mechanism [38,46,50]. Typically, release from adaptation occurs following presentation of a stimulus that does not repeat features or information to which a neuron is sensitive. Therefore, repetition suppression is more likely to reveal stimulus selectivity rather than novelty detection [49], and may provide a measure of neural tuning. For example, in the IT cortex, repetition suppression is maintained even when the location or the size of the presented stimulus is varied, suggesting that the tuning of IT neurons is invariant to both stimulus location and size [51].

While intrinsic adaptation effects can be used to infer neural tuning, as with all neural measures, it is not always possible to isolate intrinsic adaptation from inherited effects. As discussed in detail in other reviews [17,19], inherited adaptation from upstream brain regions can contaminate intrinsic adaptation and compromise the accuracy with which neural selectivity can be localized. For example, direction selectivity can emerge in area V4 when adaptation in MT affects the balance of the received inputs [52]. In the visual stream, the relative contribution of inherited and intrinsic adaptation can be determined by exploiting the difference in the receptive field size of neurons across the processing hierarchy. Once the contribution of inherited effects have been accounted for intrinsic adaptation to visual motion can appear altogether absent in area MT if the time-lag between the test and adapting stimulus is too large [53]. Critically, however, when the appropriate time interval between the test and adapting stimulus is employed, intrinsic adaptation in MT is revealed [54] and the magnitude of suppression effects shows positive correlation with the neurons' tuning curve [55]. This shows that adaptation can be used to measure neural selectivity when appropriate experimental parameters are chosen.

In other brain regions, inherited adaptation effects appear to have little or no influence. For example, neural tuning in area IT does not appear to be affected by adaptation *per se* [56,57] and adaptation to face stimuli is observed selectively in fusiform face area (FFA) despite the expectation of orientation specific adaptation in upstream areas [58]. Overall, functional selectivity may be carefully inferred using neural adaptation, when the possibility of inherited effects are acknowledged within the context of the neural circuit.

## 3. Repetition suppression in fMRI

**Figure 3.**
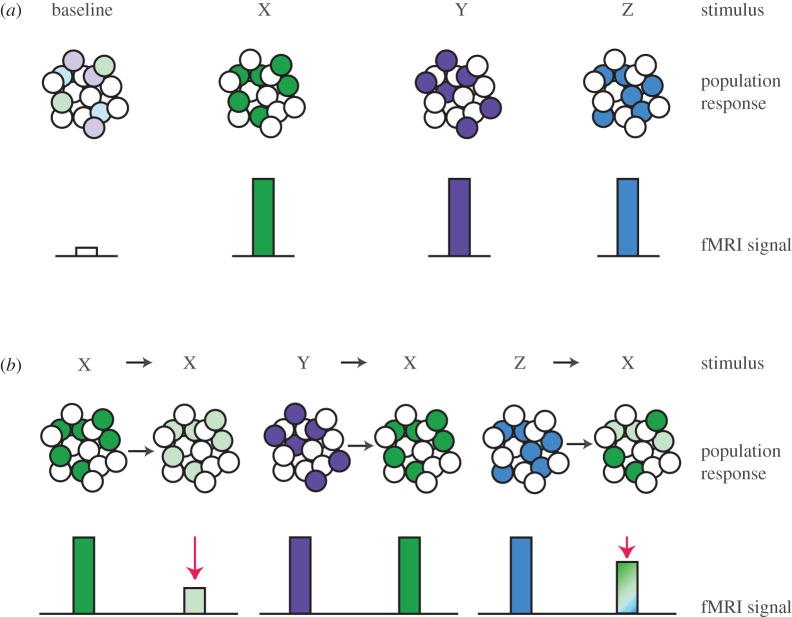
Schematic illustration of the principle underlying fMRI adaptation. (*a*) The raw BOLD signal measured in conventional fMRI paradigms provides only a measure of the mean activity of the population of neurons within a given voxel. In this example, the raw BOLD signal in response to stimuli X, Y and Z is the same, because the average neural activity within the voxel is comparable for all three stimuli. The raw BOLD signal alone is therefore invariant to the relationship between representations of stimuli X, Y and Z. (*b*) In fMRI adaptation paradigms, the relationship between different stimulus representations X, Y and Z may be indirectly measured. If stimulus X is preceded by stimulus X (X→X), then the fMRI signal in areas encoding features particular to stimulus X is suppressed. If stimulus X is preceded by stimulus Y (Y→X), the response to stimulus X should not show any suppression, as the representations for X and Y are not overlapping. If stimulus X is preceded by stimulus Z (Z→X), the response in areas encoding the features that are shared between X and Z should show suppression which scales with the amount of overlap between representations of X and Z.

Taking advantage of this neural phenomenon, repetition suppression can also be observed using fMRI, thereby providing a means to access the information content of neurons in the human brain. To measure repetition suppression using fMRI, tasks have been designed to detect reduced BOLD response in situations where a stimulus or information feature is repeated (e.g. X preceded by X), relative to situations in which the preceding stimulus or information feature is different (e.g. X preceded by Y, see figure 3). fMRI repetition suppression effects were first observed in the human visual cortex. For example, it has been shown that cortical area MT (V5) adapts to stimuli moving in a single direction [59], the primary visual cortex (V1) adapts to the orientation of visual gratings, with progressively smaller adaptation to increasingly orthogonal gratings [60], and the extrastriate and IT cortices adapt to repeated presentation of an object [61].

Among these early studies, fMRI repetition suppression was also used to investigate neural representations in the lateral occipital complex (LOC), demonstrating invariance to object size, position and illumination in this brain region [62,63]. These results suggest that the LOC represents perceived shape as opposed to simple image features [63], in a similar manner to the IT cortex in the macaque [49]. Indeed, the fMRI repetition suppression effects observed in the human LOC were later found to be comparable to fMRI repetition suppression observed in the IT cortex in the macaque [64].

## 4. The relationship between the BOLD signal, single-cell firing and repetition suppression

While fMRI repetition suppression effects measured in humans reflect those measured with single-unit recording in monkeys [62,65], before considering fMRI repetition suppression a suitable tool to access the information content of neural populations it is worth considering the relationship between the BOLD response and neural firing more closely. Notably, simultaneous measurement of BOLD (4.7T), single unit, multiunit and local field potential (LFP) measurements in the primate primary visual cortex suggest that both the BOLD signal and fMRI repetition suppression effects more closely reflect LFPs as opposed to spiking activity *per se* [66,67]. The BOLD signal may therefore be best understood to reflect integrative synaptic and dendro-somatic processes within the local network [68].

However, when considering this potentially unfortunate state of affairs, it is important to remember that in the cortex intra-regional connectivity dominates over inter-regional connectivity. Indeed, most afferent inputs are received from neighbouring neurons 1–2 mm in distance away [69,70]. Therefore, even if the BOLD response is best attributed to an integrative measure of pre- or post-synaptic processes, in many instances it probably reflects functional effects within the imaged voxel. This may explain the close correspondence between recorded BOLD and electrophysiological signals in both humans and macaques (e.g. [71,72]).

Ambiguity in the spatial localization of the BOLD signal may therefore, at least in part, be mitigated by high intra-regional connectivity. However, the indirect relationship between neural firing and the BOLD signal remains pertinent when interpreting fMRI adaptation effects. The BOLD signal depends on the ratio between blood flow and oxygen metabolism, two components of the neurovascular response that are differentially modulated by adaptation: while changes in oxygen metabolism faithfully reflect neural adaptation effects in V1, blood flow adaptation is observed to a lesser extent [73]. As a consequence, haemodynamic adaptation may underestimate adaptation at the neural level.

Despite good reason to believe that the relationship between neural and fMRI adaptation is complex, recorded data nevertheless show close correspondence between fMRI repetition suppression effects in humans and those measured with single-unit recording in monkeys [62,65]. This suggests that the degree to which haemodynamic adaptation underestimates neural repetition suppression does not preclude fMRI repetition suppression as a tool to investigate brain activity at the meso-scale. Furthermore, recent evidence from the human brain shows that fMRI repetition suppression in the LOC co-occurs with a decrease in the concentration of glutamate in the same brain region [74], supporting the conclusion that the fMRI adaptation signal faithfully reflects repetition suppression at the neural circuit level. Broadly, it seems reasonable to consider fMRI repetition suppression a suitable correlate of neural suppression effects in the voxels to which suppression is localized if interpretation is executed with caution.

## 5. Mechanisms of repetition suppression

Having characterized the BOLD signal in terms of perisynaptic activity as opposed to action-potential firing rate *per se* [75], this raises questions about the underlying mechanism responsible for repetition suppression, which is still a matter of debate.

An early proposal suggested that repetition suppression reflects ‘facilitation’ in neural signal processing. Mechanistically, the facilitation model comprises a shift in the peak latency of the neural response [76], which accounts for a reduction in overall BOLD response. This model also provides a neural explanation for behavioural priming effects [77,78] which can be observed under similar conditions to repetition suppression and manifests as a change in behavioural performance in response to repeated stimulus exposure [79,80]. For example, priming can improve reaction time and accuracy for repeated stimuli [81].

Although priming effects and repetition suppression have together been observed under the same experimental conditions, establishing a causal relationship between the two has proven difficult. For example, in non-human primates when repetition suppression and priming are observed under the same conditions, the two effects do not correlate [57]. In humans, although some studies have found a positive relationship between priming and repetition suppression [82,83], the literature is littered with inconsistencies. While one study showed that both fMRI repetition suppression and behavioural priming are disrupted with application of trans-magnetic stimulation (TMS) to the left frontal cortex [84], others have failed to find evidence for a positive relationship [85,86]. One possible explanation for the discrepancies is that although repetition suppression and behavioural priming often co-occur, repetition suppression may not necessarily causally underlie behavioural priming. Indeed, single-unit recordings in both humans and non-human primates show that repetition does not lead to faster neural responses or narrower tuning curves [56,87–90]. This suggests that the ‘facilitation’ model does not suffice as a mechanistic explanation for repetition suppression.

Single-unit data have inspired alternative mechanistic accounts for repetition suppression. For example, the ‘sharpening’ model proposed that repetition affects the selectivity or sparseness of the neural response [49,91,92]. Under this model, stimulus repetition leads to attenuation in neurons that are less selective and less well tuned, while highly selective neurons continue to respond. Although a sharpening effect has been observed when previously novel stimuli become highly familiar [88,93], this model fails to explain suppression effects observed following less familiar repetitions. Indeed, in response to repetition, single-unit measurements show that the greatest attenuation is observed in the most selective neurons as opposed to the least selective neurons [49,57,94,95].

These single-unit measurements in fact speak to a third hypothesis which explains repetition suppression as a fatigue effect, attributed to either reduced action-potential firing in neurons that are selective to a given stimulus [96,97], or attenuation in the efficacy of received inputs [39,96,98]. Evidence from macaque IT suggests that suppression speaks to the latter of these two possibilities, attenuation in the received inputs [65]. IT neurons that respond equally to two different stimuli (e.g. stimuli A and B) suppress to presentation of *A* followed by *B*, but this repetition suppression effect is not as substantial as that observed following repeated presentation of stimulus *A*. This result implies that repetition suppression of a single cell cannot be entirely action-potential dependent, and must also, at least partly, reflect the attenuation of received inputs. Not all neurons selective to stimuli A and B therefore show cross-stimulus adaptation in proportion to neural selectivity [65]. Notably, however this does not necessarily compromise the ability to use fMRI adaptation to index neural selectivity within a voxel because much of the received input comes from other neurons within the voxel. A relevant question for future investigation concerns whether, with an unbiased selection of neurons, average suppression is equal to average selectivity across the population. However, even with the data provided in [65], it is likely that with the appropriate controls, bulk suppression effects can provide information about population selectivity.

Mechanisms for repetition suppression derived from single-unit measurements can therefore be used to guide interpretation of fMRI repetition suppression effects. However, by imaging the whole brain at once, human brain imaging provides an opportunity to test alternative explanations for repetition suppression, most notably the hypothesis that repetition suppression can be accounted for by predictive coding [20,77,99]. Inspired by models of perceptual inference, predictive coding describes how the brain anticipates upcoming events and generates an error signal when predictions are violated [99,100]. ‘Top-down’ predictions are therefore received from downstream brain regions and iteratively matched against ‘bottom-up’ evidence processed within the cortical hierarchy. The mismatch between the prediction and the evidence provides an index for expectation or surprise, and constitutes a prediction-error signal. Assuming that repeated stimuli are predictable, the prediction-error signal is eliminated with repetition, attenuating the cortical response.

Theories relating repetition suppression to predictive coding motivated Summerfield and co-workers to test whether fMRI repetition suppression in FFA was modulated by the frequency at which face stimuli are repeated [101]. They observed greater repetition suppression effects when the probability of encountering a repeated trial was high (75% of trials) compared with when it was low (25% of trials). This result suggests that repetition suppression reflects a reduction in perceptual prediction error, a consequence of repeated stimuli becoming expected. This result has since been replicated using EEG [102], and when using a range of other stimuli, including auditory tones [103,104], simple shapes [105], voices [106], somatosensory stimulation [107] and objects [108]. Expectation suppression can also be observed using single-unit recording, where neurons in IT cortex show larger responses when sequences violate transition rules between visual stimuli that were learned during training [109]. These results appeal to a long line of literature showing heightened responses in scalp-evoked potentials and neuroimaging measurements when stimuli are unexpected or novel [110–113].

Although these results appear to suggest a common mechanism for repetition suppression and stimulus expectation, there is increasing evidence to suggest that these two phenomena are in fact mediated by distinct processes. Firstly, in macaque IT, expectation suppression can be observed with single-unit and LFP measurements when learned transition statistics are violated, but repetition suppression is not modulated by expectation [114]. Similarly, in humans, expectation does not modulate repetition suppression in LOC when repetition frequency is more subtly modulated (60%:40% repetition frequency) [115]. Therefore, expectation suppression is not necessarily observed in concert with repetition suppression.

Secondly, when attention is explicitly modulated in an fMRI study, expectation suppression is only observed when participants are attending [116], while repetition suppression is observed even when attention is diverted away.

**Figure 4.**
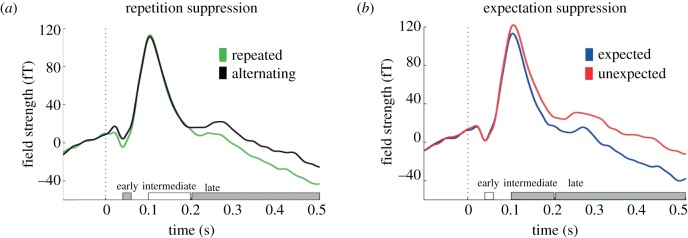
Repetition suppression and expectation suppression occur at different times in cortical processing. (*a*) Repetition of an auditory tone results in reduced neural signal during the early time bin following stimulus onset (green: repeated; black: alternating). (*b*) Expectation of an auditory tone results in reduced neural signal during intermediate time bins following stimulus onset (blue: expected; red: unexpected). Both plots: both repetition and expectation of an auditory tone give reduced neural signal during late time bins following stimulus onset. Time is denoted along the *x*-axis and periods during which a significant effect was observed are shown in grey’. The average auditory evoked response measured using MEG is shown on the *y*-axis. Adapted from [103].

Thirdly, when auditory stimulus repetition and expectation are orthogonally manipulated, auditory evoked potentials measured using MEG show repetition suppression 40–60 ms after the tone, and expectation suppression 100–200 ms after the tone (figure 4, [103]). The difference in timing between repetition and expectation suppression effects suggests that these phenomena may be mediated by two distinct processes. Within a predictive coding framework, it has been suggested that these two processes may correspond to prediction-error signals at different levels of the cortical hierarchy [103].

Human brain imaging has therefore provided insight into the mechanism underlying repetition suppression, characterizing the phenomenon within an elegant theoretical framework. This complements data from invasive animal recording which explains how repetition suppression may be biologically realized. Importantly, if concerns over localization are kept in mind and stimulus expectations and attention appropriately controlled, fMRI repetition suppression signals can be predicted irrespective of the precise underlying mechanism. Therefore, fMRI adaptation can be applied to investigate the nature of neural representations despite ambiguity in the underlying biophysical mechanism.

## 6. Repetition enhancement—how should we interpret it?

Repetition of a stimulus feature reliably leads to suppression in the neural signal, but there have also been reports of repetition *enhancement* in both single-unit recording [38,95] and fMRI [117,118]. In single-unit recordings, enhancement effects are usually observed as a smaller fraction of recorded neurons [36,38,39,42,49,55]. Similarly, in the imaging literature enhancement effects are reported less frequently than suppression effects.

By directly comparing repetition suppression with enhancement, it has been shown that enhancement effects tend to coexist within the same cortical regions as suppression, but predominantly occur within different voxels, with distinct connectivity profiles [119]. Voxels that predominately display repetition suppression preferentially correlate with activity in early brain regions and show earlier responses, while those voxels that display repetition enhancement receive information from less specific brain regions and respond later. This suggests that repetition suppression and enhancement may play distinct functional roles. Within a predictive coding framework, it has been suggested that these phenomena may map onto the prediction-error and prediction signals, respectively [119].

The underlying mechanism for repetition enhancement does, however, remains elusive, despite attempts to establish which cognitive variables lead to enhancement effects [120]. One possibility is that repetition enhancement is observed when inhibitory normalization signals are disinhibited [121,122]. Such release from inhibition has been observed in neurons in the visual cortex when a stimulus falls within the inhibitory surround of a neuron's receptive field. For example, in V1 repeated exposure to a large visual grating, covering both the centre and surround of a cell's receptive field can give rise to repetition enhancement in neurons with orientation preferences similar to the adapting orientation while smaller gratings give rise to repetition suppression [122]. Repetition enhancement by disinhibition critically depends on the local circuit connectivity and the relationship between the adapting stimulus and a neurons receptive field. While it is possible to discern these features in the visual system [121,122], attributing enhancement effects to disinhibition may be more challenging in higher cognitive areas where neural dynamics may arise from a more complex interplay between excitatory and inhibitory activity.

A second possibility is that repetition enhancement is an intrinsic biophysical feature of the neural response which is simply less permissive than repetition suppression. In support of this second suggestion, one recent study has shown that when the principal whisker of an anaesthetized rat is repeatedly stimulated, 30% of the adapting neurons in the barrel cortex show significant enhancement during the first few hundred milliseconds after adaptation [45]. This post-adaptation enhancement effect can be accounted for by delayed recovery of inhibition relative to excitation [45], which is precisely balanced at rest. This explanation is consistent with observations mentioned above which show that repetition suppression and enhancement coexist within the same cortical regions with enhancement delayed relative to suppression.

Although repetition suppression may reduce sensitivity to fMRI adaptation effects by cancelling suppression effects that occur within the same voxel, relative to suppression repetition enhancement occurs only in a small proportion of neurons [39,40,43,49,50,63]. While it is therefore important to keep interactions between excitatory and inhibitory parts of a neural circuitry in mind, repetition enhancement is unlikely to preclude the use of repetition suppression for inferring neural selectivity.

## 7. Repetition suppression as a tool for indexing neural computations in the human brain

While early studies predominantly used repetition suppression as a tool to study sensory processing in the human brain, the technique has since been used to measure more abstract neural representations such as number representations [123,124]. Furthermore, by combining fMRI repetition suppression with careful experimental design, investigators have also begun to use fMRI repetition suppression to provide mechanistic insight into neuronal computations that subserve complex human cognition. Here, we review a subset of these studies to provide a brief and non-exhaustive overview of the modern use of fMRI repetition suppression.

Firstly, repetition suppression can be used to infer representational overlap by assessing the relative suppression between two *different* stimuli. For example, neurons that contribute to the representations of both stimuli X and Z should show suppression to presentation of stimulus X preceded by Z (figure 3*b*). By contrast, suppression should not be observed when X is preceded by a stimulus Y, if stimulus Y activates a non-overlapping neural representation (figure 3*b*). Using repetition suppression to index the relative representational overlap of different stimuli in this manner can be described as ‘cross-stimulus adaptation’. This phenomenon is comparable with multivariate ‘cross-stimulus decoding’ techniques, where a classifier is trained on one set of stimuli, and tested on a different set of stimuli which are associated with or share a feature with the first set [125–127], or where the relative representational similarity of two associated stimuli is assessed using correlational measures [128,129].

At the single neuron level, cross-stimulus adaptation has been observed in neurons sensitive to two different stimuli in macaque area IT [63]. As discussed above, this effect does not occur in proportion to the neuron's response to each stimulus, as has been assumed in some models of repetition suppression [124], but is instead best explained by the similarity between the adaptor and test stimuli and by the strength of response to the adaptor stimulus [56,130]. Given that synaptic fatigue likely accounts for repetition suppression effects, cross-stimulus adaptation between two stimuli may be considered a function of the shared input or number of synapses common to processing the two stimuli [65].

Information regarding representational overlap cannot easily be inferred from the raw BOLD signal measured in conventional fMRI paradigms where only the mean response to a stimulus is assessed (figure 3*a*). However, by taking advantage of cross-stimulus adaptation, it becomes possible to index associative memories in the human brain [131]. As memory formation increases the strength of cortical connections between associated cell-assemblies [132], the increase in representational overlap can be indexed using cross-stimulus suppression. Therefore, by contrasting the BOLD response to consecutive presentation of two associated stimuli with the BOLD response to two unrelated stimuli, the representational overlap of neural representations measured using cross-stimulus adaptation can be used as a putative index for associative memory [131] (figure 3*b*). For example, after two stimuli are repeatedly imagined together, plasticity between the supporting neural representations can be assessed [133], and ongoing plasticity between a stimulus and reward representation can be tracked over time [134].

**Figure 5.**
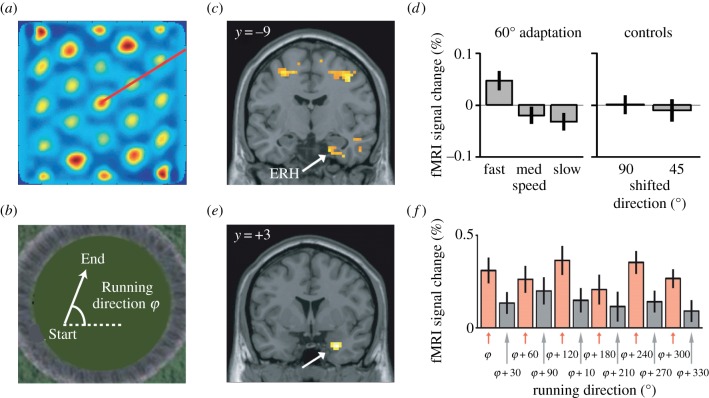
Using repetition suppression to measure grid cells in the human entorhinal cortex. (*a*) Spatial autocorrelogram of a typical grid cell in the rat entorhinal cortex, constructed from the single-unit firing rates within a box shaped environment. The grid cell's firing fields are arranged symmetrically at an angle of 60°. (*b*) fMRI adaptation was measured as a function of the angle between running directions. (*c*) Speed-dependent adaptation can be observed within the entorhinal cortex (ERH) when participants run at 60° from their previous direction. (*d*) The speed-dependent adaptation effect was specific to running angles of 60° and was not observed at running angles of 90° or 45°. (*e*) The same brain region showed a modulation of the raw activity consistent with a sixfold rotational symmetry. (*f*) Visualization of the sixfold rotational symmetry with running direction in the raw fMRI signal extracted from the entorhinal cortex. Adapted from [135].

Assessing repetition suppression between different stimuli can also be used to index *neural computations* that underlie cognitive processing. For example, fMRI repetition suppression can be used to infer the mechanism responsible for the construction of a new neural representation [133]. It can also be used to measure computations previously observed in animal models. One particularly striking example is the investigation of grid cells in the human brain. Grid cells were first discovered in the rodent entorhinal cortex, and are characterized by hexagonally arranged firing fields, which allow spatial knowledge to be organized into a map (figure 5*a*, [136]). Remarkably, the orientation of grid cells are aligned which can be exploited using fMRI repetition suppression in the human brain. When subjects navigate through a virtual environment (figure 5*b*), the entorhinal cortex, medial PFC, parietal cortex and temporal cortex show suppression as a function of running direction, modulated by running speed (here shown for the entorhinal cortex, figure 5*c*) [135]. Crucially, this adaptation effect is selective to a running direction of 60° (figure 5*d*), consistent with the predicted population response of grid cells and corresponding sixfold rotational symmetry of the raw BOLD signal (figure 5*e*,*f*) and the predicted population response of grid cells.

fMRI repetition suppression also brings a new level of mechanistic experimentation to social neuroscience. In addition to providing the first evidence for mirror neurons in the human IT cortex [137,138], fMRI repetition suppression has been used to index neural computations that allow social influence to affect the choices we make. For example, it was used to demonstrate that neural representation of the similar, but not dissimilar, traits of others overlap with the representation of our own traits [139]. Furthermore, repetition suppression can be used to show that the prediction errors caused by learning about another person's preferences can increase the representational overlap of value representations for self and other, which in turn predicts changes in behavioural preference [140]. Without representational techniques, such complex social mechanisms are difficult to infer in situations where single-unit data cannot be collected.

Together these studies illustrate how fMRI repetition suppression can be used to assess representational overlap between neural representations, which can provide a measure for associative memories and complex computations that underlie cognitive processes in the human brain.

## 8. Experimental design for fMRI repetition suppression experiments

When using fMRI repetition suppression to infer neural representations and computations in the human brain it is critical to employ an appropriate experimental design. Typically, event-related rather than block designs are used, to allow repetition suppression to be measured flexibly while controlling for the potential confounding effect of expectation suppression (figure 4), fluctuations in attention, and changes in baseline BOLD signal between sessions. The relative expectation of experiencing a given trial may be minimized by ensuring that each trial type of interest is presented equally often in a fully randomized manner. It is important to note that a trial here is defined as a stimulus transition (e.g. trial type 1: X preceded by X, trial type 2: X preceded by Y; figure 3*b*) which are typically modelled separately within a general linear model (GLM). The magnitude of repetition suppression may then be quantified by contrasting the BOLD response measured in adapting trials (e.g. X preceded by X) against the BOLD response measured in non-adapting trials (e.g. X preceded by Y).

To optimize the sensitivity of an event-related design for detection of repetition suppression, a number of additional factors must be considered. Firstly, of particular importance is the time-lag between the initial presentation of a stimulus and its repeat. In single-unit recordings, repetition sensitive neurons show a reduction in suppression as a function of repetition time-lag, with an upper limit for the majority of neurons of the order of seconds to minutes (for example, TE, perirhinal and entorhinal neurons, see figure 2, [36]). Indeed, only a small proportion of neurons show suppression effects following repetition at longer time-lags [141], leading to the suggestion that lag-sensitive repetition suppression can provide a recency trace, a measure of the relative familiarity of a stimulus [142]. Consistent with these single-cell observations, fMRI measures are also more sensitive to repetition suppression when there is a short time-lag between initial and adapting stimuli [143], or when there are few intervening stimuli between each repetition [144]. Notably, the upper limit for the fMRI repetition time-lag is typically of the order of seconds [143,144], somewhat lower than that typically observed in single-unit data [142].

**Figure 6.**
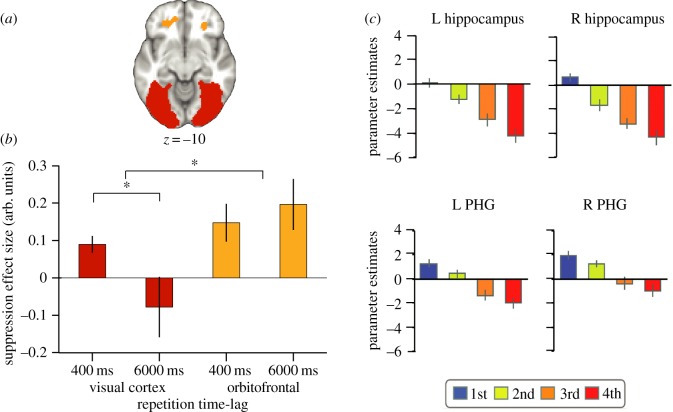
Reptition time-lag and exposure: factors to consider when designing an fMRI repetition suppression experiment. (*a*) regions of interest in the visual cortex and orbitofrontal cortex from which repetition suppression was measured, adapted from [145]. (*b*) An interaction was observed between the brain regions shown in (*a*) and the time-lag across which repetition suppression was observed. In both the visual cortex and OFC, suppression was observed if a stimulus was repeated after 400 ms. However, only the OFC showed repetition suppression if a stimulus was repeated after 6000 ms, suggesting a difference between anterior and posterior brain regions, adapted from [145]. (*c*) Repetition suppression in the left (L) and right (R) hippocampus and the parahippocampal gyrus (PHG) declines linearly as a function of the number of presentations or repetitions of a stimulus, adapted from [147].

The appropriate choice of fMRI repetition time-lag may depend on the precise brain region of interest. Indeed, as the time-lag between an adapting and a test stimulus is increased, repetition suppression effects appear to be sustained for longer in anterior compared with posterior brain regions [143,145,146]. This anterior–posterior dissociation has been illustrated by direct comparison between suppression in visual cortex to repeated visual stimuli and suppression in lateral orbitofrontal cortex to repeated stimulus-reward associations [130]. While suppression effects in the visual cortex were observed at short (400 ms) but not long (6000 ms) repetition time-lags, suppression effects in the orbitofrontal cortex were observed at both short and long time-lags (figure 6*a*,*b*). This interaction between repetition time-lag and brain region is consistent with reports of robust orientation adaptation in V1 at short time lags, of the order of a few hundred milliseconds [148,149], but not long time lags, of the order of seconds [150].

The appropriate time-lag for a given brain region is likely to be determined by the neural dynamics and recurrent processing of the region in question. While neurons in primate and rodent orbitofrontal cortex typically show sustained responses, holding information in ‘working memory’ [151], much shorter responses are observed in visual cortex and other more posterior brain regions [38]. Furthermore, the size of an fMRI adaptation effect may also depend on the duration of the adapting stimulus, whereby longer stimulus presentations result in more pronounced adaptation effects, particularly in early visual areas [152]. The optimal repetition time-lag and stimulus duration for obtaining fMRI suppression effects may therefore be determined by how long neural representations are typically sustained within the adapting brain region in question. It is therefore advisable to choose these parameters carefully by referring to previously published repetition suppression effects in the brain region of interest. In addition, when making inferences that concern the regional specificity of adaptation effects, it is important to be aware that different brain regions show varying sensitivity to different repetition time-lags. Null results should therefore not be taken as evidence for a lack of sensitivity to a stimulus feature, and inferences about regional specialization must be drawn with care.

A second factor that modulates repetition suppression effects is the relative familiarity of the stimuli. A progressive decrease in both neural firing and the fMRI response signal is observed as a function of the number of repetitions of a new stimulus [36,117,141,147,153] (figure 6*c*) or the length of exposure to the initial stimulus [154]. To avoid potential confounds relating to familiarity modulation, it is necessary to ensure that stimuli implemented in both adapting and non-adapting control trials are equally familiar. This allows repetition suppression to be measured regardless of the number of repetitions.

When designing an event-related fMRI repetition suppression study, a sufficient number of adapting trials (and equivalent number of control trials) must be included to obtain a reliable measure. To determine the appropriate number of trials, it is worth considering factors that affect the signal-to-noise ratio of the BOLD signal more generally but also the anticipated sensitivity to repetition suppression. Sensitivity to suppression is probably influenced by the degree to which the adapting feature is represented within a given voxel. Although this is difficult to estimate, the type of adapting stimuli and the functional properties of the adapting brain region in question can provide some guidance. For example, recent studies show that repetition suppression can be reliably observed in visual cortex (including V2, V3, V4, LO, MT, FFA) when 72 repetition trials are measured across two sessions [116], and in orbitofrontal cortex when 72 trials per adapting condition are measured across three sessions [145]. When repetition suppression is used to assess the online strength of orbitofrontal representations, however, it has been shown that sub-blocks with as few as nine trials can be sufficient [134].

To summarize, there are a number of different factors to consider when designing an event-related fMRI repetition suppression study. Given that suppression effects are modulated by both recency and familiarity, it is necessary to consider the appropriate repetition time-lag and the relative familiarity of repeating and control stimuli. Furthermore, repetition suppression is likely affected by the nature of the particular adapting stimulus or computation and the intrinsic circuity of the corresponding adapting brain region. To maximize signal-to-noise in fMRI repetition suppression designs, the appropriate number of trials is therefore likely to be task dependent but may be estimated using guidance from previous studies.

## 9. Concluding remarks

In this review, we have explored both the merits and limitations of using fMRI repetition suppression as a tool to circumvent the poor spatial resolution of the BOLD signal and provide non-invasive measurements of neural representations in the human brain. Repetition suppression effects are reliably observed in single-unit recordings and likely reflect the overlap between the neural representations that support the repeated stimulus feature. When measured using fMRI, suppression effects are also observed in the BOLD signal, despite the non-trivial and complex relationship between neural repetition suppression effects and BOLD adaptation. Although the potential limitations of this approach are important to bear in mind, fMRI repetition suppression may nevertheless be used to provide access to neural representations in humans at the meso-scale. This may allow associative memories and neural computations to be indexed. Along with other representational fMRI measures, such as MVPA, fMRI repetition suppression may therefore be used as a tool to investigate neural mechanisms that underlie higher cognitive function, including those that may not be amenable to direct measurement in animals.
